# Modulatory effects of extract of *Heinsia crinita* against fructose/streptozotocin-induced oxidative stress in diabetic rat models

**DOI:** 10.1016/j.heliyon.2023.e21308

**Published:** 2023-10-31

**Authors:** Iwara A. Iwara, Eve O. Mboso, Oju R. Ibor, Kelvin Elot, Collin Igajah, Andem A. Bassey, Ofem E. Eteng, Bob I.A. Mgbeje, Godwin O. Igile, Mbeh U. Eteng, Augustine Arukwe

**Affiliations:** a Department of Biochemistry, Faculty of Basic Medical Sciences, University of Calabar, P.M.B 1115, Calabar, Nigeria; b Department of Zoology and Environmental Biology, University of Calabar, P.M.B 1115, Calabar, Nigeria; c Department of Biology, Norwegian University of Science and Technology (NTNU), Høgskoleringen 5, N-7491, Trondheim, Norway

**Keywords:** *Heinsia crinita*, Lipid peroxidation, Oxidative stress, Diabetes, Molecular docking, Antioxidants

## Abstract

Oxidative stress plays a crucial role in the development of type 2 diabetes and the associated microvascular and cardiovascular complications. In the study, we have investigated the effects of *Heinsia crinita* (*H. crinita*) extracts on lipid peroxidation and oxidative stress responses using diabetic rats. Type 2 diabetes was induced with 10 % fructose/40 mg/kg body weight streptozotocin (STZ). *H. crinita* extract was administered at 200 and 400 mg/kg body weight twice daily for 21 days, in addition to metformin (MET: 500 mg/kg body weight) control. Molecular docking analysis was performed to determine the binding affinity of *H. crinita* extracts to the DNA binding domains of peroxisome proliferator-activated receptor (Ppar) and retinoid x receptor (Rxr) protein crystal structures, showing different binding affinities for putative active compounds from the plant. Fasting blood glucose (FBG), body and organ weight changes were determined showing that *H. crinita* extract induced an anti-hyperglycemic effect in the treated animals, with changes (either decrease or increase) in liver and kidney weights. A decrease in mRNA expression of peroxisome proliferator-activated receptors (*ppar*), sterol regulatory element-binding protein 1 (*srebp-1c*), liver x-receptor (*lxr*), retinoid x receptors (*rxr*), cytochrome p45041 (*cyp4a1*) and acyl-CoA oxidase (*acox1*) in diabetic animals were observed, compared to the control. A dose-specific decrease or increase in antioxidant enzymes (superoxide dismutase: SOD, catalase: CAT, reduced glutathione: GSH, glutathione peroxidase: GPx) transcripts and activity levels were also observed. We also observed exposure-specific decrease or increase of malondialdehyde (MDA) levels. Our data suggested that *H. crinita* extract possesses protective effects against diabetes-induced oxidative stress. These effects might be attributed to their binding and activation of nuclear receptors, indicating their cellular mode of action that is comparable to MET.

## Highlights

• *Heinsia crinita* produced putative anti-diabetic effects comparable to that of metformin.
• Active *Heinsia crinita* compounds bound to nuclear receptors with different binding affinities.
• *Heinsia crinita* extract induced anti-hyperglycemic effect by elevating fasting blood glucose levels.
• *Heinsia crinita* extract produced dose-specific alteration of oxidative stress responses.
• *Heinsia crinita* extract produced dose-specific alteration of transcript expression patterns.

## 1 Introduction

Diabetes mellitus (DM) is one of the oldest human diseases and a leading global health problem, with a prevalence of 463 million and expected to rise to 548 million by the year 2045. Type 2 DM accounts for >90 % of all diabetes cases and is said to be a leading cause of death in people between 50 and 70 years old [1]. This global health problem requires an urgent need to understand the underlying mechanisms of DM pathophysiology [2]. It has been proposed that diabetic complications such as retinopathy, cataracts, neuropathy, atherosclerosis, nephropathy, embryopathy, and delayed wound healing are initiated or activated through a common mechanism of free radical formation, resulting in the production of a variety of reactive oxygen species (ROS), reactive nitrogen species (RNS), and increased oxidative stress [3]. In addition, the provoked stress causes insulin resistance and altered gene expression [3].

Due to the numerous negative side effects of synthetic drug therapies, the focus of diabetes therapy is shifting from synthetic drugs to medicinal plants [4]. In addition to the negligible therapeutically observed side effects, medicinal plants are relatively inexpensive and easy to obtain for relatively less affluent rural communities. Currently, there is an upward trend in the use of polyphenol-rich medicinal plants to treat and manage various disease conditions and pathologies [5]. For this purpose, *H. crinita* has been shown to exhibit hypoglycemic, hepato-protective, and nephron-protective properties in albino Wistar rats with alloxan-induced type 1 diabetes [6]. Previously, Mgbeje and coworkers [7] revealed that extracts from *H. crinita* produced antidiabetic properties and protected against diabetes-induced electrolyte imbalance and hematological disorders. In addition, the presence of bioactive components with established antihyperglycemic, hypocholesterolemia, hypolipidemic, and antioxidant effects and possible antidiabetic effects have also been reported [8]. The diabetic rat model induced by fructose and streptozotocin replicates the characteristics of both insulin resistance (fructose) and β-cell destruction (streptozotocin). This makes it a pertinent model, for studying type 2 diabetes. *H. crinita* extract may potentially exert hypoglycemic effects by enhancing insulin sensitivity and promoting glucose uptake, thereby addressing a key underlying factor in diabetes-related oxidative stress. However, there is no information concerning the underlying molecular mechanism of the reported biological activity of this plant. Thus, this study was designed to investigate the effects of extract from this tropical plant species (*H. crinita)* on the molecular, physiological, and cellular responses related to oxidative stress of fructose/streptozotocin (STZ)-induced diabetes using Wistar rat as animal model.

## 2 Materials and methods

### 2.1 Chemicals

All chemicals and solvents used were of analytical grade. Distilled water was prepared using a laboratory-grade stainless steel benchtop still (Ningbo, China). Glassware of various types and sizes used (including beakers, graduated cylinders, Erlenmeyer flasks, separating funnels, and Bächner funnels were all S Pyrex products (North America). Other materials used were blenders (Kenwood USA, Model Blend X-Pro), retort stands with clamps, spatulas, Well cloth, filter paper (Whatman #1 with a pore size of 0.45), generic water bath (model HH-S6 China), Topton digital electronic balance (China, model ATX124). Primer pair sequences were purchased from Sigma-Aldrich Oslo, Norway. The RNA isolation kit was purchased from Zymo Research Corporation, Irvine, CA USA, iScriptTM cDNA synthesis kit, iTaq DNA polymerase, dNTPmix, and iTaqTM SYBR® Green supermix with ROX were purchased from Bio-Rad Laboratories (Hercules, CA, USA).

### 2.2 Plant collection, extraction, and fractionation

About 1 kg of *H. crinita* fresh leaves were purchased from Watt market in Calabar (Calabar, South Local Government area: LGA), Cross River State, Nigeria. The leaves were thoroughly washed and rinsed under a running tap and distilled water, respectively, and then drained for excess water. Thereafter, the leaves were spread sparsely and allowed to dry at room temperature and then pulverized into fine particles using a manual hand blender. The powdered *H. crinita* leaf sample (2756 g) was extracted by complete soaking in 90 % absolute ethanol for 48 h with intermittent agitation and gyration, a process known as cold maceration [9]. After 48 h, the solution was filtered twice with a chess cloth to remove large leaf particles and then with filter paper to remove finely ground particles. The collected filtrate (extract) was concentrated using a thermoregulated water bath at mild temperatures (35–40 °C) until complete dryness, leaving a paste (crude extract) of about 15.4 g.

Components in the crude extract were separated by liquid-liquid fractionation using methanol as solvent. The active substances were classified according to their affinity for the solvent. A phase difference resulting from the immiscibility of the solvent aided separation with a separation funnel. The phases were collected and concentrated to complete dryness using a thermoregulated water bath at 35–40 °C, to produce a paste.

### 2.3 Gas chromatography-mass spectrometry (GC-MS) analysis

The identification of the fractionated samples was performed using GC-MS-QP2010 plus (SHIMADZU-JAPAN) which consisted of an AOC-20i autosampler and a gas chromatograph interfaced with a mass spectrometer. Test conditions were as follows: fused silica capillary column (Rastek RT x 5 Ms; 30 m × 0.25 mm ID x 0.25 μm film thickness) consisting of 5 % diphenyl, 95 % dimethylpolysiloxane; column oven temperature maintained at 80 °C, injection temperature at 250 °C; split injection mode; pressure of 108 kpa; total flow of 6.2 mL/min at 1 mL/min; column flow of 1.58 mL/min; split ratio of 1.0 and solvent separation time of 2.50 min. Mass spectra were taken at a start time of 3 min and an end time of 27 min; while the ACQ mode scan was performed at event time (0.50 s) with a scan speed of 1250 m/s.

### 2.4 Molecular docking analysis

Fifteen (15) identified bioactive compounds in *H. crinita* extracts were used as ligands for the docking analysis. The chemical structures of these compounds and that of the co-crystallized (standard ligand) proteins were obtained from the PubChem compound database (https://pubchem.ncbi.nlm.nih.gov). The three-dimensional chemical structures of the protein molecules Ppar-γ and Rxr were retrieved from the protein databank (www.rcsb.org). The structural data files (SDF) of the ligands were downloaded and subjected to molecular docking with Ppar-γ and Rxr protein targets. The proteins were prepared using Chimera 1.14 by removal of non-essential water molecules, non-standard proteins, and the addition of hydrogen and charges. The SDF formats of the ligands were prepared and converted to the protein data bank, partial charge (Q), and atom type (T) (PDBQT) file using the PyRx tool to generate atomic coordinates, and energy was minimized by optimization using the optimization algorithm at the force field. The ligands were docked to the protein targets and the binding affinities were determined using Autodock Vina from PyRX. Molecular interactions between proteins and ligands were viewed with Chimera 1.14 and Discovery Studio 2020.

### 2.5 Handling of animals

The present study was approved by the Faculty of Basic Medical Sciences Animal Ethics Committee of the University of Calabar with approval number 149BCM3021. Six (6) weeks old 40 female albino Wistar rats weighing 150–200 g were obtained from the animal facility of the College of Medical Sciences, University of Calabar. The experimental animals were distributed for the study as follows: normal control (NC) administered water *ad libitium*, diabetic control (DC) administered water *ad libitium*, and diabetic groups were treated with 200 and 400 mg/kg b.w of methanol fraction of *H. crinita* (MHc) extract dissolved in water.

All rats were housed in wooden cages at 25 °C, 50–60 % relative humidity, and 12:12 h photoperiod. They were allowed to acclimatize to a diet and water *ad libitium* for one week before being used for the experiment. Oral administration of the extract and MET (500 mg/kg b.w) was performed twice daily for 21 days via gastric intubation. The doses of extract administered were chosen based on our preliminary study that established a lethal dose (LD) of 5000 mg/kg b.w that was supposedly to kill 50 % of the test animal in 24 h (LD_50_). Although no mortalities were observed during the 24-h period, the body weights and fasting blood glucose (FBG) levels of the experimental animals were monitored.

### 2.6 Induction of type 2 diabetes

Diets were formulated to contain 10 % fructose dissolved in distilled water and mixed with rat chow purchased from Vital Feed, in Jos, Nigeria and fed to the study animals daily for 14 days as described by Wilson and Md [10]. The rats were deprived of food (i.e. fasted) overnight and a single dose of 40 mg/kg streptozotocin dissolved in 0.1 M sodium citrate buffer (pH 4.4) was administered by intraperitoneal injection. The FBG levels were measured using blood obtained by tail puncture at 72 h after injection using a standby plus glucometer. Animals with a FBG of 7.8 mmol/l or 180 mg/dl were considered diabetic and included in the study.

### 2.7 Blood and tissue sampling

On day 21, the animals were fasted overnight by removing food with constant access to water (for about 14 h) on the last day of the experiment, before the collection of the tissue samples. Before sacrifice, the test animals were anesthetized with ketamine/xylazine at 70mg/7 mg, respectively, through intraperitoneal injection. Liver and kidney tissues were removed and blotted with Whatman filter paper to clean the excess blood and weighed. Thereafter, a portion of the tissue was sliced and suspended in RNALater for gene expression analysis. The remaining tissue was homogenized using mortar and pestle in 0.1 M phosphate buffer. The homogenate was centrifuged at 3000 rpm for 10 min and the supernatant was collected and used for biochemical analysis. The percentage (%) change in FBG was calculated. The liver and kidney were weighed using a precision multifunctional balance (APOLLO/GF-A, Australia), and their relative weights to total body weight were calculated as liver- and kidney somatic index (LSI and KSI, respectively).

### 2.8 Biochemical analysis

SOD activity was determined in μmol/mg as the auto-oxidative inhibition of epinephrine by measuring the increase in absorbance at 480 nm as described previously [11]. In brief, the reaction mixture (3 ml) contained 2.95 ml of 0.05 M sodium carbonate buffer (pH 10.2), 200 ml of tissue homogenate, and 300 ml of epinephrine. The reaction was initiated using 0.005 N HCl. The reference cuvette contained 2.95 ml buffer, 300 ml of the substrate (epinephrine), and 200 ml of water. Enzyme activity was calculated by measuring the change in absorbance at 480 nm for 5 min.

CAT activity was measured in μmol/mg a previously described method [12]. This method is based on the reduction of dichromate/acetic acid reagent into chromic acetate in the presence of hydrogen peroxide (H_2_O_2_). Briefly, the reaction was initiated at 37 °C by adding 0.5 ml of 0.2 M H_2_O_2_ to the reaction mixture containing 1 ml of 0.1 M sodium phosphate buffer (pH 7.4) and 0.5 ml of tissue homogenate in a total reaction volume of 2 ml. The reaction was stopped after 1 min by adding 2 ml of dichromate/acetic acid reagent (5 % potassium dichromate with acetic acid; 1:3 v/v). The blank was prepared by the addition of 1 ml of buffer and 2 ml of dichromate acetic acid. The test and blank tubes were then heated for 10 min in a boiling water bath to develop a green color. After cooling to room temperature, the intensity was measured at 570 nm against the blank. The CAT-specific activity was expressed in μmoles H_2_O_2_ decomposed/min/mg protein.

Reduced GSH content of tissues as non-protein sulfhydryl was estimated according to previously described method [13]. In brief, to the homogenate, a 10 % trichloroacetic acid (TCA) was added and centrifuged. Thereafter, 1 ml of supernatant was treated with 0.5 ml of Ellman's reagent (19.8 mg of 5, 5-dithiobisnitro benzoic acid (DTNB) in 100 ml of 0.1 % sodium nitrate) and 3 ml of phosphate buffer (0.2 M, pH 8). The absorbance was read at 412 nm.

The GPx activity was determined in μmol/mg as described previously [14]. In brief, 0.2 ml Tris-buffer, EDTA, sodium azide (10 mM), and 0.2 ml of triplicate sample homogenate were mixed. To this, 0.2 ml of GSH followed by 0.1 ml of H_2_O_2_ were added and incubated at 37 °C for 10 min. The reaction was stopped by the addition of 0.5 ml 10 % TCA. The tubes were centrifuged at 2000 rpm and 1 ml of the supernatant was transferred to a fresh tube. I ml of distilled water (blank) and the GSH standard (200 μg/ml) at a concentration range of 5–20 μg were also taken in separate tubes. To all the above tubes, 4 ml of disodium hydrogen phosphate (0.3 M) and 0.5 ml of DTNB -5-5′dithio bis 2 nitrobenzoic acid substrate (DTNB - 0.6 mM in 1 % trisodium citrate) were added. The color developed was read at 420 nm immediately, against the blank.

The MDA was used as a measure of lipid peroxidation and was determined using the method of Buege and Aust [15]. In brief, 1.0 ml of the sample supernatant was added to 2 ml of (1:1:1 ratio) TCA-TBA HCl reagent (thiobarbituric acid 0.37 %, 0.24 N HCL and 15 % TCA), tricarboxylic acid-thiobarbituric acid-hydrochloric acid reagent and boiled at 100 °C for 15 min and allowed to cool at room temperature. Flocculent materials were removed by centrifuging at 3000 rpm for 10 min. The supernatant was removed, and the absorbance read at 532 nm against a blank. MDA was calculated using the molar extinction coefficient for MDA-TBA-the complex of 1.56 × 105 M − 1 cm-1.

### 2.9 RNA isolation, cDNA synthesis, and qPCR analysis

The liver tissue was thawed, removed from RNAlater, and pad dried. Total RNA was isolated by using a Direct-zol RNA Miniprep Kit following the manufacturer's protocol (Zymo Research Corporation, Irvine, CA USA). Using 1 μg RNA, complementary DNA (cDNA) strands were generated using a combination of oligo (dT) and random hexamer primers from iScript cDNA synthesis kit, as described by the manufacturer (Bio-Rad, Oslo Norway).

Real-time (quantitative) polymerase chain reaction (real-time PCR) was performed with gene-specific primers (Supplementary information (SI) Table S1) using the Mx3000 P real-time PCR system (Stratagene, La Jolla, CA). Each 20 μL DNA amplification reaction contained 13 μL of Light cycler SYBR Green supermix (2 × ), 5 μL cDNA, and 1 μL each of the forward and reverse primers. The three-step real-time PCR program included an enzyme activation step at 95 °C (10 min) and 40 cycles of 95 °C (15 s), 60 °C (15 s) and 72 °C (15 s).

### 2.10 Statistical analysis

Results were analyzed for statistical significance by one-way ANOVA (Analysis of variance) with post hoc Tukey test at (p < 0.05) using the Prism GraphPad 8.2.1 (GraphPad Software, La Jolla, USA). All data were expressed as mean ± SEM (n = 8 replicates).

## 3 Results

### 3.1 GC-MS analysis of *H. crinita* leaf extract

**Fig. 1:**
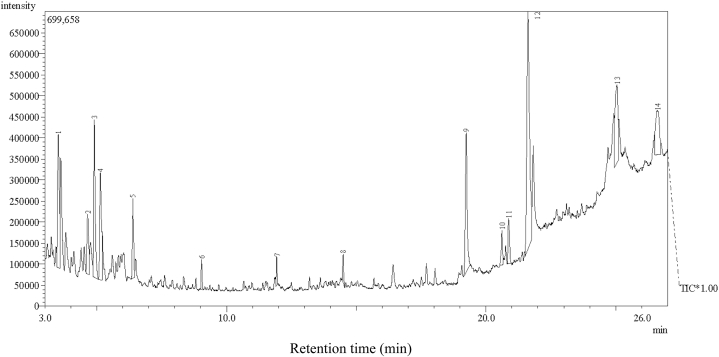
Chromatogram of methanol fraction of *Heinsia crinita* (HC).

GC-MS analysis of the methanol fraction of H. crinita revealed the presence of 14 dominant compounds (Table 1) and an associated chromatogram (Fig. 1). Among the observed 14 compounds, oleic acid was the most abundant (27 %), followed by palmitic acid (11.1 %), nonane (9.9 %), a-limonene diepoxide (9.8 %), 1,2,3-trimethylbenzene (8.1 %), and oxalic acid (7.4 %). There were other compounds observed at trace concentrations with non-significant percentage abundance.

**Table 1:** Compound composition of the methanol fraction of *Heinsia crinita* leaf extract.

Peak	Retention time^a^	Area^b^	Height^b^	Compound name
1	3.514	9.92	11.05	Ethylbenzene
2	4.652	4.39	4.95	1-Ethyl-2-methylbenzene
3	4.909	9.85	13.08	Nonane
4	5.141	8.05	8.91	1,2,3-Trimethyl benzene
5	6.395	4.13	6.62	2,7-Dimethyloctane
6	9.042	1.11	2.48	2,4-Dimethylhexane
7	11.937	0.99	2.42	3,3-Dimethylhexane
8	14.495	1.17	2.34	2,8-Dimethylundecane
9	19.236	11.05	11.6	Palmitic acid
10	19.236	1.99	2.89	Cyclopropanepentanoic acid
11	19.236	2.98	3.73	13-Docosenoic acid
12	21.618	27.13	19.74	Oleic acid
13	25.044	9.82	6.49	α-Limonene diepoxide
14	26.599	7.42	3.68	Oxalic acid

a Values are given in minutes.

b Values are given in percentage (%).

### 3.2 Molecular docking analysis

**Fig. 2:**
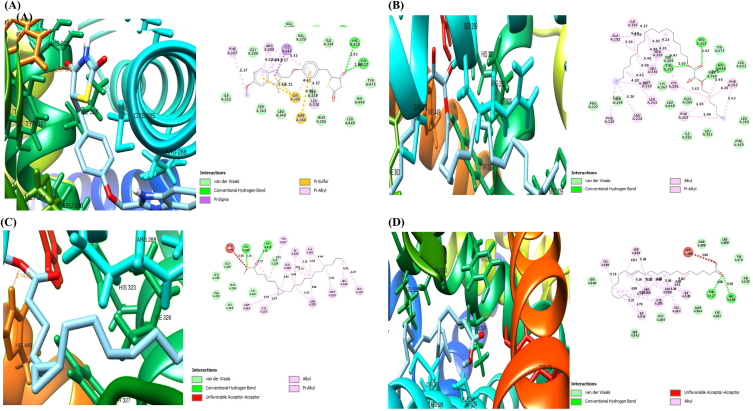
The three and two-dimensional (3D and 2D) view of the molecular interactions of amino acid residues of Ppar-γ with rivoglitazone (A), 13-docosenoic acid (B), oxalic acid (C), and cyclopropane pentanoic acid (D).

**Fig. 3:**
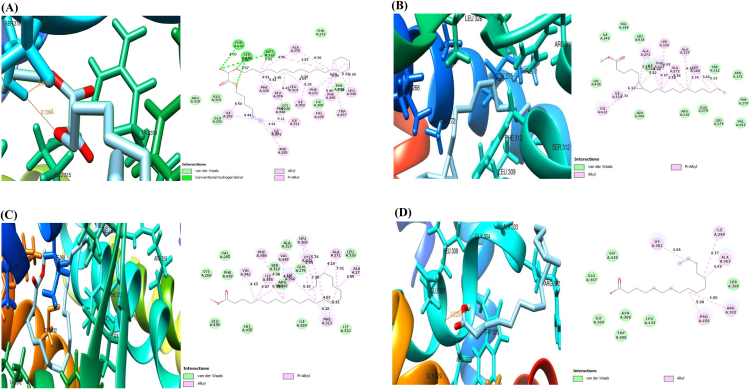
The three and two-dimensional (3D and 2D) view of the molecular interactions of amino acid residues of Rxr with [E]-3-[4-hydroxy-3-(3,5,5,8,8-pentamethyl-6, 7-dihydronaphthalen-2-yl)phenyl]prop-2-enoic acid (A), cyclopropanepentanoic acid (B), 13-docosenoic acid (C), and palmitic acid (D).

The 3D and 2D structures and docking results of Ppar-γ, Rxr, co-crystalized compounds, and compounds from H. crinita are shown in Fig. 2, Fig. 3 (A–D, respectively) and Table 2. The results showed compounds from HC demonstrated varying degrees of binding affinities for Ppar-γ and Rxr by the change in Gibbs free energy (ΔG). Against Rxr, four compounds from H. crinita namely-cyclopropanepentanoic acid (−7.5 kcal/mol), 13-docosenoic acid (−6.8 kcal/mol), palmitic acid, and 1-ethyl-2-methylbenzene (−6.2 kcal/mol) showed binding affinities that are closely related to the standard co-crystallized ligand (E)-3-[4-hydroxy-3-(3,5,5,8,8-pentamethyl-6, 7-dihydronaphthalen-2-yl)phenyl]prop-2-enoic acid (−7.4 kcal/mol). Oxalic acid (−6.8 kcal/mol), cyclopropanepentanoic acid (−6.6 kcal/mol), 13-docosenoic acid (−6.1 kcal/mol) interacted with Ppar-γ with their docking scores closely related to rivoglitazone (−8.8 kcal/mol). These compounds interacted with the amino acids in the binding pockets in a similar manner as their standard co-crystallized ligands.

**Table 2:** Binding affinities (ΔG in kcal/mol) of Rxr and Ppar-γ with bioactive components of *Heinsia crinita*.

S/N	Compound name	CID	Rxr	Ppar-γ
1	13-docosenoic acid	5363109	−6.8	−6.1
2	2,8-dimethylundecane	519384	−5.7	−5.7
3	Oleic acid	445639	−4.5	−6
4	Limonene dioxide	232703	−6	−5.2
5	1-ethyl-2-methylbenzene	11903	−6.2	−5.3
6	3,3-dimethylhexane	11233	−5.1	−4.8
7	1,2,3-trimethylbenzene	10686	−6	−5.1
8	Palmitic acid	985	−6.2	−5.8
9	Cyclopropanepentanoic acid	91691232	−7.5	−6.6
10	Nonane	8141	−5	−4.9
11	Ehtylbenezen	7500	−6	−5.5
12	Oxalic acid	6420369	−4.6	−6.8
13	2,7-dimethyloctane	14070	−5.6	−5.7
14	(E)-3-[4-hydroxy-3-(3,5,5,8,8-pentamethyl-6, 7- dihydronaphthalen-2-yl)phenyl]prop-2-enoic acid	44566110	−7.4	–
15	Rivoglitazone	3055168	-	−8.8

### 3.3 Effects of *H. crinita* and MET on FBG glucose levels

**Fig. 4:**
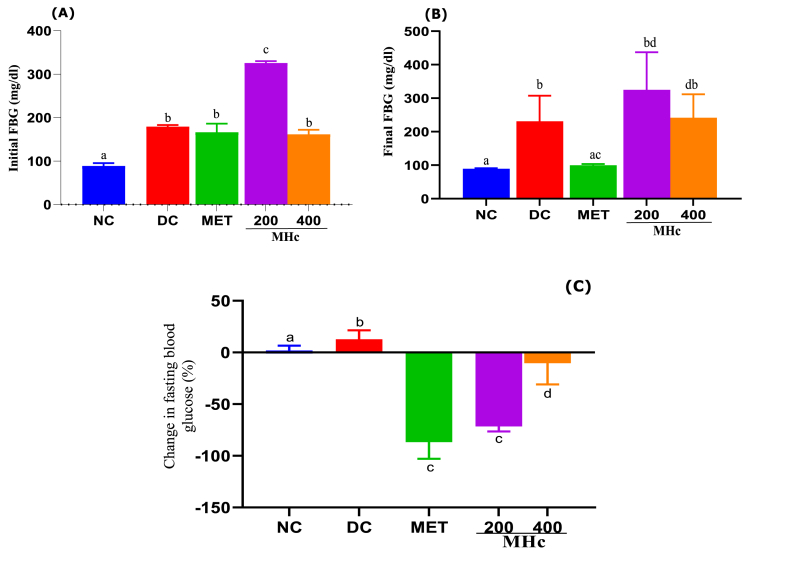
Initial and final blood glucose (A and B, respectively), and percentage (%) change in fasting blood glucose (C) after 21 days administration of *Heinsia crinita* (HC) extract and metformin in diabetic rat models. NC: Normal control, DC: Diabetic control, MET: Metformin, MHc: Methanol HC. Data are given as mean ± standard error of the mean (SEM) of n = 8. Different letters (a, b, c, d and e) denote significant difference between treated groups at p < 0.05.

The result of the FBG is presented in Fig. 4, showing that the FBG of the diabetic control group during the initial treatment period increased significantly (p < 0.05), compared to the NC group (Fig. 4A). Treatment of the diabetic groups showed a significant (p < 0.05) decrease in the concentration of final FBG for the MET and MHc400 groups, compared to the DC and MHc200 groups (Fig. 4B), while the MHc200 exposure produced significant reduction in FBG compared to the DC group, but still significantly higher than MHc400 and MET groups (Fig. 4B). The percentage change in FBG is shown in Fig. 4C, demonstrates a validation of the results on the initial and final FBG as earlier presented. The result showed a significant (p < 0.05) percentage decrease in the groups treated with MET, MHc200, and MHc400, compared to the DC and NC control groups.

### 3.4 Effects of *H. crinita* and MET on organ weight

The weight of the liver and kidney tissues of experimental rats treated with *H. crinita* extract and MET are presented in Table 3, Table 4, respectively. For the liver, no significant (p > 0.05) changes were observed in the liver weight and LSI of the DC group compared to the NC group (Table 3). On the other hand, a significant (p < 0.05) decrease in liver weight was observed in the MHc 200 treated group, compared to other experimental groups. A significant increase in LSI was observed in the MET-treated group, compared to the DC and NC groups (Table 3). For the kidney, no significant (p > 0.05) difference in KSI was observed in the diabetic control group compared to the normal control group (Table 4). However, treatment with the *H. crinita* extract and MET showed a significant (p < 0.05) increase in kidney weight in all treated groups (MET, MHc 200, and MHc 400), compared to the DC and NC groups (Table 4).

**Table 3:** Absolute liver weight and liver somatic index (LSI), and oxidative stress parameters in treated animals.

	Liver						
	Weight (g)	LSI (%)	SOD (μmol/mg protein)	CAT (μmol/mg protein)	GPx (μmol/mg protein)	GSH (μg/ml)	MDA (μmol/L)
NC	4.6 ± 0.7^a^	0.03 ± 0.002^a^	38.9 ± 5^a^	57.6 ± 1.7^a^	4.6 ± 0.4^a^	27.7 ± 1.2^a^	6.1 ± 0.4^a^
DC	4.93 ± 0.42^a^	0.033 ± 0.003^a^	79 ± 4.3^b^	100.6 ± 3^b^	5.6 ± 0.4^a^	32.6 ± 1^b^	14.1 ± 1.2^a^
MET	5.23 ± 0.4^a^	0.04 ± 0.01^ab^	53.6 ± 3.6^b^	68.8 ± 3.2^a^	4.7 ± 0.3^a^	28.8 ± 0.9^a^	10.5 ± 0.6^a^
MHC 200	4 ± 0.33^b^	0.034 ± 0.002^a^	56.2 ± 2.84^b^	81.74 ± 4.7^b^	5 ± 0.3^a^	31.2 ± 1.2^b^	12 ± 1^a^
MHC 400	5.3 ± 0.4^a^	0.036 ± 0.003^a^	52 ± 3.34^b^	75.6 ± 2.8^a^	4.8 ± 0.3^a^	29.5 ± 0.8^b^	10.8 ± 0.4^a^

SOD: superoxide dismutase, CAT: Catalase. GPx: Glutathione peroxidase, GSH: Glutathione,MDA: Malondialdehyde.

**Table 4:** Absolute kidney weight and kidney somatic index (KSI), and oxidative stress parameters in treated animals.

	Kidney						
	Weight (g)	KSI (%)	SOD (μmol/mg protein)	CAT (μmol/mg protein)	GPx (μmol/mg protein)	GSH (μg/ml)	MDA (μmol/L)
NC	0.93 ± 0.14^a^	0.01 ± 0.0004^a^	27.1 ± 3^a^	29.6 ± 2.64	4.44 ± 0.2^a^	24.3 ± 1^a^	4.2 ± 0.1^a^
DC	0.83 ± 0.05^a^	0.006 ± 0.001^a^	44.7 ± 3.6^b^	50.2 ± 2.5^b^	4.7 ± 0.2^a^	28.7 ± 0.9^b^	5.2 ± 0.54^a^
MET	1 ± 0.2^a^	0.007 ± 0.001^b^	44.8 ± 3^b^	35.94 ± 1.4^a^	4.3 ± 0.3^a^	25.3 ± 0.8^a^	5 ± 0.5^a^
MHC 200	1.03 ± 0.2^a^	0.009 ± 0.002^b^	47 ± 2.4^b^	44.3 ± 2^b^	4.6 ± 0.3^a^	27.5 ± 1^b^	4.93 ± 0.3^a^
MHC 400	1.1 ± 0.2^a^	0.007 ± 0.001^b^	43.5 ± 3^b^	39.8 ± 1.5^b^	4.6 ± 0.3^a^	25.94 ± 0.7^b^	5 ± 0.3^a^

SOD: superoxide dismutase, CAT: Catalase. GPx: Glutathione peroxidase, GSH: Glutathione.

MDA: Malondialdehyde.

### 3.5 Effects of *H. crinita* and MET on mRNA expression

**Fig. 5:**
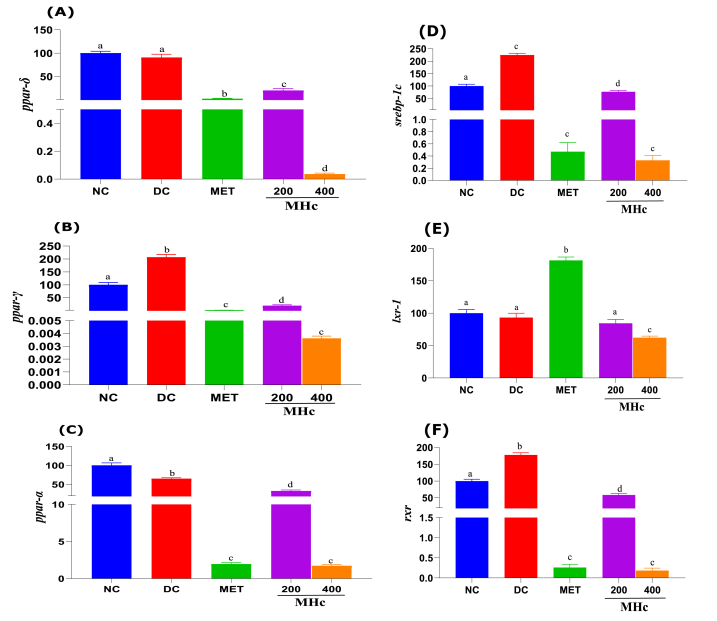
Transcript (mRNA) expression patterns of *ppar*s (α, γ and δ: A-C, respectively), *lxr*, *rxr and Srebp-1c* (D-F, respectively) in the liver of rats after 21 days administration of *Heinsia crinita* (HC) extract and metformin in diabetic rat models. NC: Normal control, DC: Diabetic control, MET: Metformin, and MHc: Methanol HC. Data are given as mean ± standard error of the mean (SEM) of n = 8. Different letters (a, b, c, d and e) denote significant difference between treated groups at p < 0.05.

The liver expression of *ppar* transcript isoforms (α, γ, and δ) after exposure to *H. crinita* extract, normal, and diabetic control groups is shown in Fig. 5. A significant (p < 0.05) decrease in the expression of *ppar*-d was observed in the MET, MHc 200, and MHc 400 treated groups, compared with the DC and NC groups (Fig. 5A). For *ppar*-γ, a significant (p < 0.05) increase was observed in the DC group and a decrease in the MET, MHc 200, and MHc 400 treated groups compared to the NC group (Fig. 5B). Treatment with *H. crinita* extract and MET produced significant (p < 0.05) decreases in the expression of *ppar*-α, compared to both the NC and DC groups (Fig. 5C). The MHc 400 treatment regime severely decreased the expression of *ppar*-α, γ and δ, compared to MHc 200 and MET (although these treatments decreased *ppar* expressions).

The transcript expression patterns of *srebp-1c, lxr-1*, and *rxr* in the liver tissues of rats treated with extracts of *H. crinita* and MET are shown in Fig. 5D, E, and F, respectively. A significant (p < 0.05) increase in *srebp-1c* expression was observed in the DC group, compared to the NC group (Fig. 5D), while the treatment with MET, MHc 200, and 400 produced significant (p < 0.05) decreases in *srebp-1c* (more so for MET and MHc 400), compared to the DC and NC groups (Fig. 5D). For *lxr-1*, a significant (p < 0.05) increase in transcript expression was observed in MET-treated rats, while a significant decrease was only observed in the MHc 400 treated rats, compared with the NC and DC groups (Fig. 5E). The *rxr* transcript expression followed a comparable pattern to the *srebp-1c,* showing a significant (p < 0.05) increase in expression in the DC group, compared to the NC group (Fig. 5F), while the treatment with MET, MHc 200, and 400 produced significant (p < 0.05) decreases of *rxr* (more so for MET and MHc 400) compared to the DC and NC groups (Fig. 5F).

**Fig. 6:**
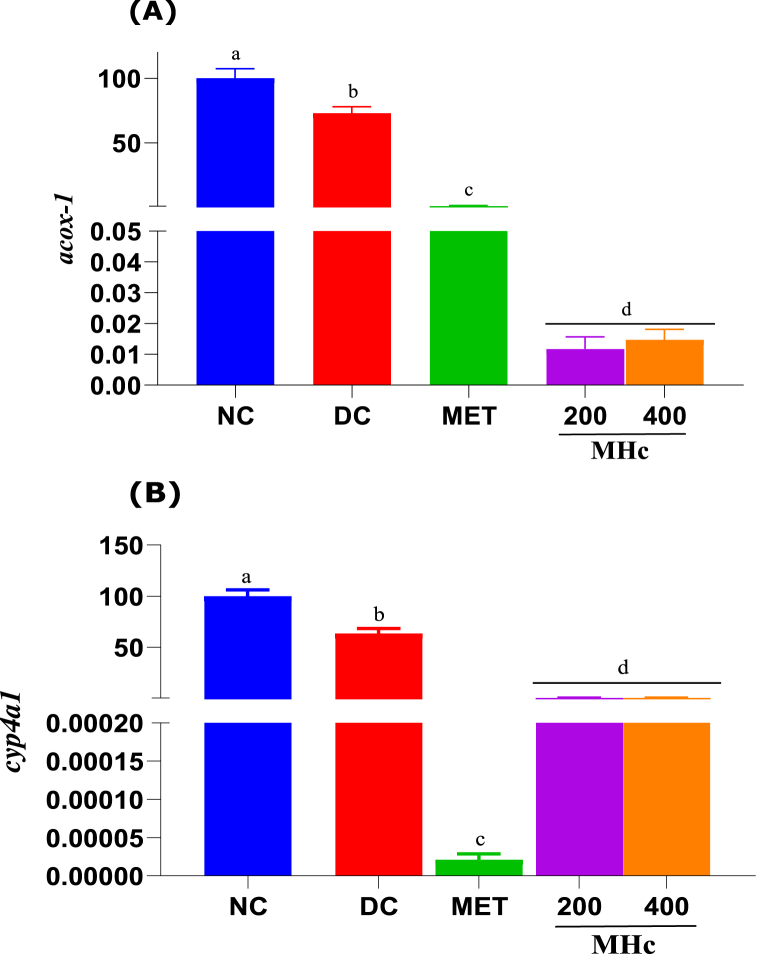
Expression of *cyp4a1* (A) and *acox-1* (B) transcripts (mRNA) in the liver of rats after 21 days administration of *Heinsia crinita* (HC) extract and metformin in diabetic rat models. NC: Normal control, DC: Diabetic control, MET: Metformin, and MHc: Methanol HC. Data are given as mean ± standard error of the mean (SEM) of n = 8. Different letters (a, b, c, d and e) denote significant difference between treated groups at p < 0.05.

The transcript expression of *acox-1* and *cyp4a1* is shown in Fig. 6. The *acox-1* transcript showed significant (p < 0.05) decreases in the DC, MET, MHc 200, and 400 groups (more so in the MHc groups), compared to the NC group. (Fig. 6A). For *cyp4a1*, significant (p < 0.05) decreases in the DC, MET, MHc 200, and 400 groups (more so in the MET groups) were observed compared to the NC group. (Fig. 6B).

### 3.6 Effects of *H. crinita* extracts and MET on oxidative stress responses

The hepatic activity levels for SOD, CAT, and GPx are shown in Table 3. SOD activity significantly (p < 0.05) increased in all exposure groups, compared to the NC group. For the CAT activity, all exposure groups produced an increase in the activity level (albeit significant only in the DC and MHc groups), compared to the NC group. No exposure-related changes were observed in GPx activity. The hepatic levels of GSH and MDA are shown in Table 4. All exposure groups produced significant (p < 0.05) increases in the GSH level (except the MET-treated group), compared to the NC group, and no exposure-related changes were observed in MDA levels (Table 3).

In the kidney, the activity levels for SOD, CAT, and GPx are shown in Table 4. Similar to the liver, SOD activity significantly (p < 0.05) increased in all exposure groups, compared to the NC group. For CAT, all exposure groups produced a significant (p < 0.05) increase in the activity level (except the MET group), compared to the NC group (Table 4). No exposure-related changes were observed in GPx activity. The kidney levels of GSH and MDA are shown in Table 4. All exposure groups produced significant (p < 0.05) increase in the GSH level (except the Met-treated group), compared to the NC group, and no exposure-related changes were observed in kidney MDA levels (Table 4).

## 4 Discussion

Due to the roles of lipid peroxidation and oxidative stress in the pathogenesis of type 2 diabetes and associated complications, we have investigated the possible molecular mechanisms of action of *H. crinita* in modulating the fructose/streptozotocin-induced lipid peroxidation and oxidative stress in diabetic rats. Our results showed that *H. crinita* contains some bioactive compounds (oleic acid, palmitic acid, nonane, α-limonene diepoxide, 1,2,3-trimethylbenzene, and oxalic acid) with potential anti-cancer, anti-inflammatory, anti-microbial, anti-diabetic, and antilipidemic effects [[16], [17], [18], [19], [20]]. *In silico* analysis of these bioactive compounds produced different binding affinities for the target proteins (Ppar and Rxr). These findings were validated *in vivo,* showing direct nuclear receptor activation of genes involved in fatty acid, glucose, and lipid homeostasis and being comparable to the mechanism of MET. Hence, these findings may indicate that *H. crinita* is a promising therapeutic agent in the management of DM and its related medical complications.

In this study, we performed GC-MS analysis on *H. crinita* and observed the presence of 14 bioactive compounds, with oleic acid having the highest percentage abundance, followed by palmitic acid, and oxalic acid having the lowest percentage abundance. Consistent with our findings, it has been previously suggested that these bioactive compounds possess anti-diabetic and anti-lipidemic activities [[16], [17], [18], [19], [20]]. Molecular docking of Rxr with *H. crinita* ligands showed that four compounds - cyclopropanepentanoic acid, 13-docosenoic acid, palmitic acid, and 1-ethyl-2-methylbenzene interacted strongly with the Rxr protein. The ligands occupied a different binding site from that of the co-crystallized compound, suggesting a possible allosteric interaction. The observed interactions were stabilized by weak bonds such as van der Waal forces and alkyl and pi-alkyl bonds, and the amino acid residues occupying these regions for the *H. crinita* ligands differed from those of the standard ligand. Further, for the Ppar docking, we observed that three compounds, oxalic acid, cyclopropanepentanoic acid, 13-docosenoic acid, bind favorably to the receptor, occupying the same binding site with the standard ligand, and are also stabilized with conventional hydrogen, van der Waal forces, alkyl, binding pi-alkyl, sulfur and sigma bonds. Other reports on molecular studies of Ppar proteins have shown that the binding of an agonist and simultaneous binding of an antagonist to the active site of Ppar isoforms does not affect the specificity of the domain, as it switches the mode of action of the receptor from induction to repression [21].

Streptozotocin (STZ) is an antibiotic that causes the destruction of pancreatic islet cells and is widely used experimentally to model type 1 DM [22]. Herein, streptozotocin with 10 % fructose was used to induce type 2 diabetes, a multifactorial disease associated with various complications such as obesity, hypertriglyceridemia, impaired glucose tolerance, and increased insulin resistance, with a high financial burden to the healthcare economy [23]. Herein, we observed an increase in fasting blood glucose in the DC group per the induction of T2DM using 10 % fructose/streptozotocin. Treatment with MET and the extract produced a significant decrease in the concentration of blood glucose. However, 21 days of treatment with MET and *H. crinita* extract modulated the observed increase in blood glucose level, thus, indicating potential anti-diabetic activity of this plant. Consistent with our findings, it has been reported that *H. crinita* extract possesses anti-diabetic activity in rat models [7]. Furthermore, no changes in FBG were recorded for the DC, indicating the presence of prolonged hyperglycemia leading to DM [24]. We observed a significant decrease in FBG in the MET exposure group, with a corresponding similar decrease in the MHc 400 group confirming a similarity in activity. This observation is particularly interesting and may suggest that the *H. crinita* extract slightly mimics the activity of MET in the control of DM and related complications.

Under normal conditions, the liver and kidney act as a source of endogenous antioxidants to scavenge ROS produced by xenobiotics [25]. However, if the levels of these antioxidants are overwhelmed by free radicals, or when these organs are under pressure from oxidative stress influence, damage to these organs would increase, leading to inflammation [26]. Hepatic and renal hypertrophy are some of the complications associated with diabetes with a reported frequency of 40–70 % [27]. Treatment with *H. crinita* extract caused a decrease in liver weight and an increase in the liver somatic index in the MHc200 treated group. Kidney weight and KSI increased in all treated groups. The observed changes in liver and kidney weights (while minimal) demonstrated the potential adaptive effects of *H. crinita* to maintain liver tissue integrity and ameliorate potential liver and kidney hypertrophy associated with a diabetic condition. However, these should be further investigated using a differently designed experimental approach.

Ppar, Rxr and Lxr isoforms are nuclear receptors (NRs) that control the expression of a variety of genes involved in vital biological processes including fatty acid, glucose, and lipid metabolism [28]. Lxr and *ppar* are members of nuclear receptors that form obligate heterodimers with Rxr [28]. *Ppar* play an important role in regulating cell differentiation, embryonic development, cellular and whole-body metabolism, inflammation, and tumorigenesis in higher organisms [29]. They play a crucial role in maintaining the functions and homeostasis of metabolic organs such as muscle, adipose tissue, and liver [30] and regulate lipid homeostasis by controlling the balance between the use and storage of long fatty acids. They are not only activated by fatty acids and their derivatives but also by many natural substances of plant and marine origin [31]. A decrease in *ppar* mRNA expression was observed in the diabetic groups treated with *H. crinita* extract and MET. This observed decrease can be attributed to the anti-diabetic activity of this plant [6] by preventing the breakdown of triacylglycerol from adipose tissue for energy in diabetes, leading to the release of free fatty acids and thereby inactivating/inhibiting the expression of *ppars*. Also, the observed effects of *H. crinita* were comparable to MET. Previously, Raheleh and coworkers [32] reported a strong correlation between the activation of *ppar isoforms* by medicinal plants. Elsewhere, Erfani et al. [33] showed that over 200 bioactive compounds, particularly flavonoids, present in *H. crinita* were either agonists or antagonists of *ppar* isoforms. These compounds may play integral roles in the modulation of blood glucose levels observed in the present study.

In this study, no change in *lxr* expression was observed in the NC, DC, and MHc200 experimental diabetic groups except for the MET and MHc400 groups. Previous reports have demonstrated that *lxr* plays crucial roles in the metabolism of cholesterol and hepatic induction of fatty acids and triacylglycerol biosynthesis [34,35]. This further suggests that extract of *H. crinite* at a dose level of 400 mg/kg bodyweight may have some effect on the metabolism of carbohydrates and overall metabolism of lipids as opposed to MET. According to Toshimasa et al. [36], any suitable antagonist of *ppar* and rxr may protect against complications associated with type 2 diabetes. Based on our data, the induction of diabetes with streptozotocin increased mRNA expression of *rxr* in the diabetic experimental group. Treatment with MET and *H. crinita* extract reduced *rxr* mRNA expression. The observed reduction was more pronounced in the MET and MHc 400 treated groups. This could further indicate possible similarities in the mechanism of action of *H. crinita* and MET. Furthermore, it is important to note that the decrease of some of these nuclear receptors observed in the study by MET and *H. crinita* extract, may be an indication of a possible molecular and cellular mechanisms involved in the regulation of lipid metabolism under diabetic conditions [37,38].

The *srebp*-*1c* is a transcription factor that mediates insulin effects on hepatic gene expression. It controls lipid synthesis from glucose in the liver and this process is of great importance for energy storage. *Srebp-1c* is regulated by multiple factors including insulin [39] and lxr [40]. In this study, we observed an increase in *srebp*-1c expression after the induction of diabetes, which was reversed after treatment with MET and *H. crinita* extracts at MHc400, showing comparable reduction levels of *srebp-1c* mRNA. This effect is also a possible mechanism involved in maintaining glucose homeostasis. Consistent with our observation, elevated levels of *srebp-1c* in type 2 DM state were previously reported, indicating that MET inhibits *srebp*-1c expression and prevents the development of fatty liver [41,42]. Oxidative stress status is believed to be an important factor in the development of diabetic complications [43]. Excess formation and/or deficiency in the removal of these highly reactive molecules such as ROS and RNS leads to the development of oxidative stress, which plays a cardinal role in the development of complications associated with DM [44]. In this study, the induction of diabetes with streptozotocin significantly increased the concentration of MDA (an indicator of lipid peroxidation), SOD, and CAT activity in the experimental rat, indicating a possible effect of the ROS produced in the animal and the ability of the endogenous antioxidant enzyme in the experiment to reverse these abnormalities in the diabetic condition. After treatment with *H. crinita* extract, a reversal in SOD and CAT activity and MDA levels were observed in both liver and kidney tissues, with similarities to MET. The observed effects of this plant can be related to the presence of bioactive compounds [45]. The decrease in antioxidant enzyme activity observed in this study may also be related to the downregulation of *ppars* in the liver tissue of the experimental animals. According to [46], modulation of the *ppars* pathway has been reported to exert transcriptional regulation on the expression of endogenous antioxidant enzyme mechanisms.

It was observed that the activity of *cyp4a1* was significantly decreased in all animals induced with diabetes in this study. Cytochrome P450 (Cyp450) is an enzyme that highly expressed in the liver and associated with xenobiotic metabolism [47]. Besides their role as detoxifying enzymes, they could also have potentially harmful effects as their overproduction can lead to the generation of oxidative stress through the formation of ROS in the diabetic state, which can further lead to lipid peroxidation [48]. P450s are also reported to be involved in many biochemical processes such as fatty acid metabolism [49]**.** The observed reduction in the activity of *cyp4a1* by *H. crinita* extract and MET in our study suggest a possible efficacy and mechanisms of action of these treatments and a possible protective effect against complications of oxidative stress associated with DM. However, we observed that *acox*1 mRNA expression was reduced in *H. crinita*, MET, and NC groups. The report by Zeng et al. [50] documented that acox-1 plays a crucial role in lipid metabolism and its inhibition represents a novel and effective way of treating obesity-induced metabolic disorders by enhancing mitochondrial lipid and ROS metabolism.

## 5 Conclusion

The present study has demonstrated that *H. crinita* leave extracts modulated streptozotocin/fructose-induced nuclear receptors and antioxidant enzyme activities in diabetic rats, suggesting possible mechanisms by which this plant exerts its biological activities. Further, these effects could be attributed to the presence of bioactive compounds found and reported in this tropical plant. Further, the binding of these ligands to the pockets of Ppar with significant side effects provides an interesting link to be explored for the synthesis of new binding assays with therapeutic effects. This pharmaceutical breakthrough has been sought for several years for the management of DM and its associated complications. Nevertheless, while the novelty of the present study is evident, it also has some limitations. For example, the findings from rat models may not directly translate to human responses, limiting the generalization of the results. Further, our study focused solely on the modulatory effects of *H. crinita* extract, ignoring potential interactions with other treatment protocols and physiological factors. Diabetes is a multifaceted condition, and addressing oxidative stress alone may not fully represent its complexity. All these limitations should be considered when designing further studies on the effects of this and other tropical plants.

## Data availability

All necessary data are included in our manuscript. Any other raw data associated with our manuscript will be made available on request.

## CRediT authorship contribution statement

**Iwara A. Iwara:** Writing – original draft, Formal analysis, Conceptualization. **Eve O. Mboso:** Writing – review & editing, Formal analysis. **Oju R. Ibor:** Writing – review & editing, Methodology, Investigation, Formal analysis, Conceptualization. **Kelvin Elot:** Writing – review & editing, Formal analysis. **Collin Igajah:** Writing – review & editing, Visualization, Formal analysis. **Andem A. Bassey:** Writing – review & editing, Investigation. **Ofem E. Eteng:** Writing – review & editing, Conceptualization. **Bob I.A. Mgbeje:** Writing – review & editing, Conceptualization. **Godwin O. Igile:** Writing – review & editing, Formal analysis. **Mbeh U. Eteng:** Writing – review & editing, Investigation. **Augustine Arukwe:** Writing – review & editing, Supervision, Resources, Project administration, Methodology, Investigation, Funding acquisition, Conceptualization.

## Declaration of competing interest

The authors declare that they have no known competing financial interests or personal relationships that could have appeared to influence the work reported in this paper.
