# Supplementary material for: Modulatory effects of extract of Heinsia crinita against fructose/streptozotocin-induced oxidative stress in diabetic rat models
Source: Heliyon. 2023 Oct 31;9(11):e21308. doi: 10.1016/j.heliyon.2023.e21308 (PMC10665683; doi:10.1016/j.heliyon.2023.e21308)
Supplement: Multimedia component 1 [file mmc1.docx]

**Supplementary information (SI)**

**Modulatory Effects of Extract of Heinsia crinita against Fructose/Streptozotocin-Induced Oxidative Stress in Diabetic Rat Models**

Iwara A. Iwaraa, Eve O. Mbosoa, Oju R. Ibor b,c, Kelvin Elota , Collin Igajaha, Andem A. Basseyb, Ofem E. Etenga, Bob I.A. Mgbejea, Godwin O. Igile a, Mbeh U. Etenga, Augustine Arukwec*

aDepartment of Biochemistry, Faculty of Basic Medical Sciences, University of Calabar, P.M.B 1115, Calabar, Nigeria.

bDepartment of Zoology and Environmental Biology, University of Calabar, University of Calabar, P.M.B 1115, Calabar, Nigeria.

cDepartment of Biology, Norwegian University of Science and Technology (NTNU), Høgskoleringen 5, N-7491, Trondheim, Norway

Table S2. Primer pair sequences used for transcript amplification

| **Gene name** | **Forward primer** | **Reverse primer** |
| --- | --- | --- |
| *ppar-α* | CACGGAGCTCACAGAATTT | CAGCATCCCGTCTTTGTT |
| *ppar-β* | AGCCTCAACATGGAATGTC | CGATCGCACTTCTCATACTC |
| *lxr-1* | GCTCTGCCTACATCGTGGTC | CTCATGGCCCAGCATCTT |
| *rxr* | GCAGCACTGAGGATATCAAG | CATAGTGTTTGCCTGAGGAG |
| *fasn* | CGTTTCATCAGGCCACTATAC | ACTTGGAGTTCGGGTCTT |
| *srebp-1c* | CGGCTGTTGTCTACCATAAG | CAGTGTTGCCATGGAGATAG |
| *esr1* | AGATGACTTGGAAGGCCGAA | AAGGACAAGGCAGGGCTATT |
| *cyp4a1* | GACAAGGACCTACGTGCTGA | GTTGGTGCTTAGGGTGTGTG |
| *acox-1* | AATGTTCCTGCCCACCTTGC | GCCAAACTTGGGTCCTATGTCC |
